# Neuroimmune interactions: How past lessons can inform the present and future of MS care

**DOI:** 10.1177/13524585251370884

**Published:** 2025-09-01

**Authors:** Adil Harroud, Jack P Antel

**Affiliations:** The Neuro (Montreal Neurological Institute-Hospital), Montréal, QC, Canada; Department of Neurology and Neurosurgery, McGill University, Montréal, QC, Canada; Department of Human Genetics, McGill University, Montréal, QC, Canada; The Neuro (Montreal Neurological Institute-Hospital), Montréal, QC, Canada; Department of Neurology and Neurosurgery, McGill University, Montréal, QC, Canada

**Keywords:** Neuroimmune interactions, multiple sclerosis, glial cells, oligodendrocytes, immune-mediated demyelination

## Abstract

The recognized spectrum of neuroimmune interactions has expanded significantly, with increasing relevance to the pathogenesis and course of multiple sclerosis (MS). This review examines the bidirectional nature of these interactions—how systemic immune activity can initiate or modulate central nervous system (CNS) pathology, and how CNS-derived signals may shape peripheral immune responses. Particular attention is given to lesion compartmentalization, the impact of aging and comorbidity, and the dual roles of glial and immune cells in injury and repair. By integrating recent findings from experimental models and clinical observations, we consider how these principles inform evolving therapeutic approaches in MS and related disorders. This present overview was presented in part as the Kenneth P. Johnson Memorial Presidential Lecture at the ACTRIMS Forum 2025.

## Introduction

In recent years, as summarized in Table 1, the recognized spectrum of bidirectional neuroimmune interactions has expanded significantly, providing important insights into mechanisms that may underlie the development and course of MS and related disorders. Neuroimmune interactions operate both under homeostatic and disease conditions and can contribute to processes of tissue injury as well as repair. Conversely, observations from MS, particularly in the modern therapeutic era, inform the relevance of these mechanisms to the disease itself and highlight areas requiring further investigation. Such cross-cutting feedback has been present since the recognition of MS as a clinicopathological entity by Charcot in the 1870s and the report of neurologic complications following administration of Pasteur’s neural tissue–based rabies vaccine in the 1880s.

**Table 1.** Spectrum of neuroimmune interactions.

Neuro-directed systemic immune responses (autoimmunity)
Modulation of neuroimmune interactions by genetic and environmental factors
Neuroimmune regulation within the CNS parenchyma and specialized niches ○ Glial cells as regulators and effectors of immune responses ○ Meningeal and periventricular niches ○ Target cell (oligodendrocyte/neuron) interactions with immune mediators
Neuroregulation of systemic immune compartments and peripheral organs

Reconciling basic principles of neuroimmune interactions with clinical application requires consideration of several variables, including genetic heterogeneity among patients and exposure to complex external and internal (e.g. microbiome-derived) environments. One must also account for the impact of aging and comorbid diseases associated with advancing age. Particularly relevant to the MS population are the long-term effects of therapies acting on either immune or neural compartments.

## Systemic immune injury of CNS—meeting criteria for autoimmunity

The classical observations derived from Pasteur’s rabies vaccine demonstrated that activation of immune constituents in the systemic compartment in response to neural antigens could result in inflammation within the central nervous system (CNS). This clinical disorder, now referred to as post-vaccination encephalomyelitis, and its animal model, experimental autoimmune encephalomyelitis (EAE), raise the question of what criteria must be met to define a disorder as having an “autoimmune” pathogenesis.^1^ Here, we consider whether MS and related disorders fulfill such criteria (Box 1), derived from those used in infectious disease (Koch’s postulates).

Box 1. **Defining disease mechanisms: infectious and autoimmune models.**

Infectious disease—Koch’s Postulates

• Presence of agent/immune mediator(s) at site of pathology

• Agent/immune mediator(s) present only in those with the disease

• Agent/immune mediator(s) can be used to transfer the disease

• Removal of agent/mediator(s) has therapeutic effects

Autoimmune criteria—Witebsky (1957)^2^

• Autoimmune response be recognized in the form of an autoantibody or cell-mediated immunity

• The corresponding antigen be identified

• An analogous autoimmune response be induced in an experimental animal & animal develops a similar disease

Autoimmune revisited—Rose/Bona (1993)^1^

• Direct evidence from transfer of pathogenic antibody or pathogenic T cells

• Indirect evidence based on reproduction of the autoimmune disease in experimental animals• Circumstantial evidence from clinical clues

### Antigen specificity and MS

The most widely held hypothesis in MS involves an immune response directed against a myelin constituent, although there remains no clear consensus on the identity of the target antigen. A persistent hypothesis is molecular mimicry with exogenous agents that share homology with neural proteins, with recent focus on Epstein-Barr virus (EBV).^3,4^ MS has a strong association with HLA-DR15 molecules. Wang et al. showed that HLA-DR15–restricted CD4+ T cells can cross-react with self-peptides derived from DR2a and DR2b, peptides from MS-associated microbes including Epstein-Barr virus and *Akkermansia muciniphila*, and with autoantigens.^5^ Clinical trials in MS patients vaccinated with altered peptide ligands (APLs) of myelin peptides highlighted the variability of immune responses, ranging from regulatory to inflammatory, even in response to the same antigenic challenge.^6,7^

Clinical examples of new-onset or exacerbated white matter inflammation associated with tumor necrosis factor (TNF)-blocking agents^8^ and immune checkpoint inhibitors^9^ suggest that non-specific activation or dysregulation can precipitate CNS-reactive responses. This implies that autoreactive cells may exist in the immune repertoire of the general population, independent of disease-specific antigen exposure and the significance of immune regulatory mechanisms.^10^ The antigenic specificity of oligoclonal bands (OCBs) in MS remains undefined, unlike the measles antibody response in subacute sclerosing panencephalitis.^11^ However, a recently described longitudinally stable autoantibody motif, detectable in a subset of people with MS including before symptom onset, shares homology with epitopes from several pathogens, including EBV virus, suggesting molecular mimicry as a plausible trigger.^12^

Thus, although classical autoimmune criteria (Box 1) emphasize discovery of a single disease-defining autoantigen, growing evidence—in lupus, type 1 diabetes, and other organ-directed disorders—indicates that pathogenic autoimmunity likely involves broad, evolving repertoires of antibody and T-cell specificities, a paradigm that likely applies to MS.

### Animal models of MS

EAE provides a model for the “outside-in” pathogenesis of MS—that is, a disease initiated by systemic immune responses—as opposed to “inside-out” mechanisms originating within the CNS. Extensive studies using this model have yielded important insights into the regulation of antigen-specific responses, trafficking patterns of activated cells, and their behavior upon CNS entry. With relevance to MS, the EAE model shows that immune responses are sculpted by genetic and environmental influences—such as specific-pathogen-free housing (which attenuates disease) and microbiota transplants from people with MS (which exacerbate it)^13,14^—and by aging, which modifies both disease severity and phenotype.^15,16^

Major efforts have aimed to convert EAE from its usual monophasic course into a model more reflective of progressive MS, that is, one that is resistant to therapies targeting the systemic immune system. As reviewed by Zhang et al.,^16^ middle-aged animals develop a more severe and protracted clinical course than their younger counterparts. In parallel, patients with MS show molecular and clinical evidence of accelerated aging. Atkinson et al.^17^ demonstrated that this age-dependent exacerbation and chronicity of EAE is driven by aged, non-hematopoietic, radio-resistant cells, including glia.

### Therapeutic responses in MS

Current therapies in MS are directed at the systemic immune system. Results with high-dose ablative therapies, such as high-intensity autologous bone marrow transplant, establish that systemic components underlie the clinical relapses and acute lesion formation of MS.^18^ The failure of similar therapies to halt the progressive phase once it is established indicates the involvement of additional mechanisms. Nevertheless, increasing evidence indicates that early restriction of systemic immune access to the CNS can reduce the likelihood of later progression. Long-term extension studies show that patients randomized to interferon-β from baseline had lower rates of progression and mortality than those who started after a placebo delay.^19^ Similarly, observational studies demonstrate that initiating high-efficacy therapies earlier confers long-term benefits,^20^ with clinical trials underway. These observations have shifted therapeutic strategies toward early, highly effective treatment, rather than waiting for disease severity to declare itself.

### Comparison with related disorders

In contrast to MS, its related disorders—AQP4 + neuromyelitis optica (NMO) and myelin oligodendrocyte glycoprotein antibody-associated disease (MOGAD)—meet (or nearly meet) autoimmune criteria, with identification of specific antigens that elicit both antibody and T-cell responses, as well as therapeutic responses to antigen removal or neutralization of effector pathways.^21–23^ While animal models exist for these disorders, key questions remain. They provide strong precedent for “outside-in” disease but raise additional issues, such as the relationship between clinical phenotype and the topographic distribution of the relevant antigen. Although these disorders are characterized by recurrent episodes, they are not currently thought to evolve into a clinically progressive phase.^24,25^ Subclinical neurodegeneration has been reported in NMO, particularly affecting the visual system, although its significance and underlying mechanisms remain unclear.^24^

MOGAD is now recognized to account for a substantial proportion of cases previously diagnosed as MS in children. When these cases are accounted for, rates of EBV seropositivity among pediatric-onset MS converge toward those seen in adults, reinforcing the relevance of EBV infection in MS initiation.^26^ Notably, EBV infection is not linked to either AQP4+ NMO or MOGAD, further supporting its disease-specific association with MS.^27^

## Modulation of neuroimmune interactions—environment and genetics

Long observed has been the uneven worldwide distribution of MS, with migration studies indicating that disease risk increases when migration occurs early in life.^28^ Concordance is increased—but not absolute—in monozygotic twins,^29^ and disease phenotype can vary between siblings. These data suggest contributions of both environmental and genetic factors to disease susceptibility and course. Large, population-based studies have since identified specific environmental risk factors and modifiers of disease severity, such as smoking, EBV infection, and the microbiome, all of which are linked to neuroimmune interactions either in the systemic or CNS compartments.^30,31^ Genome-wide association studies have revealed associations between MS susceptibility and a broad set of immune-related genes, in addition to the dominant effect of the HLA locus and shared risk with other autoimmune diseases.^32^ Notably, the genetic variants linked with disease progression appear to differ from those associated with susceptibility.^33^

## Intra-CNS neuroimmune interactions

That such interactions are dynamic and reflect the activation state of both immune and neural elements was recognized over a century ago, when it was shown that the prolonged survival of brain grafts in histoincompatible hosts was shortened by superimposing a donor-derived skin graft.^34^ Functional outcomes of immune-neural interactions within the CNS increasingly appear to depend on the interface regions as well as on interactions occurring within the parenchyma itself.

### Systemic to CNS migration

The clinical efficacy of anti-adhesion molecule therapies in MS and EAE supports the importance of immune cell trafficking from the periphery across the blood–brain barrier.^35^ Additional routes include the blood–cerebrospinal fluid (CSF) barrier at the choroid plexus and, more recently, direct migration through dura-associated meningeal channels.^36–38^ Cells and immune mediators accumulating in meningeal niches are increasingly implicated in the subpial demyelination and periventricular tissue damage characteristic of MS.^39^

### CNS parenchyma

Most focus has been on microglia and astrocytes, which can contribute both to injury and to protection and repair. Studies in EAE in which astrocytes were genetically ablated showed that astrocyte depletion during the acute phase worsened disease severity, suggesting a protective role. In contrast, depletion during the chronic phase reduced disease severity, implying a shift to a pathogenic role.^40^ White matter degeneration in vanishing white matter disease has been linked to toxic astrocyte responses involving mammalian Target of Rapamycin (mTOR) pathway mutations.^41^ Regarding microglia, these cells acquire multiple phenotypes in response to environmental conditions resulting in their capacity to contribute to issue injury (“pathogenic”) and conversely to recovery by removing injured tissue and stimulating myelin repair by oligodendrocyte (OL) lineage cells (“reparative”).^42^ In MS, microglial activation, such as through elevated CSF chitotriosidase (CHIT1) levels, has been shown to moderately correlate with future disability accumulation.^43^

Microglial products can impair oligodendrocyte precursor cell (OPC) differentiation but can also promote myelination.^44–46^ Loss of microglia due to congenital absence or inactivation of CSF-1 receptor underlies adult-onset leukoencephalopathy with axonal spheroids and pigmented glia (ALSP), and its model (FIRE mice) displays distinct axonal pathology.^47,48^ Advances in RNA sequencing are beginning to reveal the molecular signatures that shape microglia–astrocyte crosstalk.^49^ This axis is responsive to microbiome-derived metabolites such as tryptophan, which can reach the CNS and modulate cellular function.^50^

### Effector and target interactions

In MS, the OL–myelin unit remains the principal target of immune-mediated injury, prompting questions about mechanisms of damage and of repair—or its failure.^51^ OLs, in addition to generating myelin for saltatory conduction, also provide metabolic support to neurons. They respond to neuronal activity by altering myelin production—so-called “adaptive myelination”—providing a link between environmental input and structural response.^52^ Early-stage OLs can express immune-related molecules, including Major Histocompatibility Complex (MHC) proteins, making them both targets and potential antigen-presenting cells.^53^

Studies of MS lesions suggest that mature OLs are relatively preserved early in disease, with progressive loss over time.^54^ OPCs are often more depleted than mature OLs in active lesions, possibly reflecting their increased vulnerability. At the lesion edge, OPCs are often enriched, suggesting attempted repair.^55^ In vitro, human adult-derived OLs are more resistant to regulated cell death than pediatric-derived OLs or early-stage OPCs.^56,57^ Notably, Raine and Prineas observed minimal apoptosis among OLs in MS lesions.^58,59^

Our in vitro studies show that OL cytotoxicity requires cell–cell contact and can be mediated through MHC class I–restricted mechanisms (e.g. CD8+ T cells), or via non-MHC–dependent pathways (e.g. γδ T cells, natural killer (NK) cells, Th17 cells), often promoted by adhesion molecules and inflammatory mediators.^51,60^ Rodriguez et al.,^61^ examining acute MS biopsy samples, noted preservation of OL somata with degeneration of processes—a “dying-back” pattern also seen in early animal models.^62^ This degeneration can be induced in vitro by TNF, interferon (IFN)-γ, excitotoxins, or metabolic stress.^55^ Sustained stress (e.g. glucose and nutrient deprivation) leads to energy failure and necrotic-type death. Importantly, reversing stress can prevent this transition to cell death.^55,63^ In progressive MS, normal-appearing white matter shows signs of metabolic stress and pro-inflammatory mediator accumulation.^64^ We have shown that persistent stress granules in OLs require combined metabolic and inflammatory stimuli and are present in situ in MS brain.^65^ Related mechanisms may include misfolded proteins in OLs that can damage neighboring neurons.^66^ OL susceptibility varies by species, age, and developmental stage. Of interest are regionally enriched mediators that may account for subpial and periventricular injury in MS. Fibrinogen, a blood-derived protein, accumulates in MS lesions and inhibits OPC differentiation, although it does not impair the ability of mature OLs to ensheath nanofibers.^67,68^

### Neuroimmune-mediated protection and repair

Remyelination in MS is highly variable, influenced by age, lesion location, severity of initial damage, chronicity, and extracellular matrix composition. Immune cells can promote repair. In animal models, mice lacking adaptive immune cells show impaired recovery from injury.^69^ Microglia are critical for remyelination; their absence impairs repair, while specific microglial products promote it.^46^ Th2-derived cytokines, including insulin-like growth factor (IGF)-1, may also contribute to regenerative responses.^70^

### CNS direction of systemic compartment

Feedback from the CNS can influence the systemic immune system and peripheral organs. Myelin components have long been observed in cervical lymph nodes.^71^ Recent studies have identified transport mechanisms by which CNS-derived materials are drained into systemic circulation, including the glymphatic system, involving astrocyte regulation of perivascular flow.^72^ In animal models, injury to OLs (e.g. by diphtheria toxin or cuprizone) can lead to subsequent autoimmune encephalitis, potentially through induction of autoreactive T cells or epitope spreading.^73–75^ The autonomic nervous system, particularly the vagus nerve, may also mediate CNS-to-systemic signaling.^76,77^

## Conclusion

The interplay between neuroimmune biology and MS has advanced both fields while raising new challenges. Principles derived from neuroimmune interactions have contributed to the emergence of effective therapies for MS, while clinical observations in MS have in turn informed basic science. However, key discrepancies remain, such as the beneficial effects of TNF blockade in EAE versus its exacerbating effects in MS patients. These outcomes underscore that core pathogenic mechanisms are not universally shared across contexts. Definitive studies are needed to clarify how neuroimmune interactions contribute to disease initiation, progression, niche-related pathology, and repair. Emerging tools—including molecular profiling of disease tissue, imaging technologies, and results from therapeutic trials—offer promising avenues for advancing our understanding of neuroimmune mechanisms in MS and beyond. Lessons from MS may apply to other neurologic and autoimmune disorders that share common principles of injury and repair.
